# Scatter Irradiation of Rat Brain Triggers Sex- and Brain Region-Specific Changes in the Expression of Non-Coding RNA Fragments

**DOI:** 10.3390/epigenomes6040035

**Published:** 2022-10-12

**Authors:** Anna Fiselier, Boseon Byeon, Yaroslav Ilnytskyy, Olga Kovalchuk, Igor Kovalchuk

**Affiliations:** 1 Cumming School of Medicine, University of Calgary, Calgary, AB T2N 1N4, Canada; 2 Biomedical and Health Informatics, Computer Science Department, State University of New York, 2 S Clinton St, Syracuse, NY 13202, USA; 3 Department of Biological Sciences, University of Lethbridge, Lethbridge, AB T1K 3M4, Canada

**Keywords:** non-coding RNA, ncRNA fragments, tRFs, frontal cortex, hippocampus, cerebellum, sex-specific

## Abstract

Non-coding RNA fragments (ncRFs) are small RNA fragments processed from non-coding RNAs (ncRNAs). ncRFs have various functions and are commonly tissue-specific, and their processing is altered by exposure to stress. Information about ncRFs in the brain is scarce. Recently, we reported the brain region-specific and sex-specific expression of ncRNAs and their processing into ncRFs. Here, we analyzed the expression of ncRFs in the frontal cortex (FC), hippocampus (HIP), and cerebellum (CER) of male and female rats exposed to scatter radiation. We found multiple brain region- and sex-specific changes in response to scatter radiation. Specifically, we observed decreased miRNA expression and the increased expression of ra-ncRNA reads in HIP and CER, as well as an increased number of mtR-NA-associated reads in HIP. We also observed the appearance of sense-intronic ncRNAs—in females, in HIP and FC, and in males, in CER. In this work, we also show that tRNA-GlyGCC and tRNA-GlyCCC are most frequently processed to tRFs, in CER in females, as compared to males. An analysis of the targeted pathways revealed that tRFs and snoRFs in scatter radiation samples mapped to genes in several pathways associated with various neuronal functions. While in HIP and CER these pathways were underrepresented, in FC, they were overrepresented. Such changes may play an important role in pathologies that develop in response to scatter radiation, the effect known as “radio-brain”, and may in part explain the sex-specific differences observed in animals and humans exposed to radiation and scatter radiation.

## 1. Introduction

Non-coding RNAs (ncRNAs) are the molecules that typically do not encode any protein and have various functions, mostly around the regulation of gene expression, from the control of the chromatin structure to the regulation of transcription and translation either directly or through modifications of other RNA molecules.

Many groups of ncRNAs have tissue-specific expression [1], indicating their important role in cell growth and differentiation. In addition, ncRNAs are frequently induced by internal metabolic stresses and external environmental stresses [2,3]. ncRNAs are also altered in various diseases and can be used as biomarkers of disease progression [4,5]. Males and females differ in terms of ncRNAs expression in normally functioning cells as well as in disease pathology [6,7].

The brain is a complex organ, and its function is also known to be regulated by ncRNAs in a brain region-specific manner [8,9]. The expression of microRNAs (miRNAs) [9] and long non-coding RNAs (lnc-RNAs) [10] in the brain is also sex-specific. ncRNAs are involved in the precise regulation of temporal and special responses of the brain transcriptome to physiological and pathological stimuli [11]. miRNAs such as miR-124 are involved in the regulation of the differentiation of neural progenitor cells into neurons [12]. lncRNA Rhabdomyosarcoma 2-associated transcript (RMST) regulates neurogenesis [13]. ncRNAs such as miRNAs, lncRNAs, and circRNAs are dysregulated in various neuropsychiatric disorders such as Alzheimer’s Disease, Parkinson’s Disease, Autism, and many more (reviewed in [14]).

Recent work analyzed the expression of ncRNAs in the adrenal glands, amygdala, hippocampus (HIP), and hypothalamus of pigs. Among all ncRNAs annotated, miRNA, tRNA, piRNA, and snoRNA were found to be differently expressed in the analyzed brain regions [8]. Similarly, the frontal cortex (FC) and HIP had different levels of expression of various miRNAs in mice [15].

Another level of complexity is presented by the fact that several types of ncRNAs are also processed into RNA halves or RNA fragments (ncRFs). tRNAs, rRNA, snoRNAs, and snRNAs are among the most frequently processed ncRNAs. The mechanism of processing ncRNAs is beyond the scope of this manuscript, but it is known that various fragments, known as tRH–tRNA halves or tRF–tRNA fragments, as well as tRNA-derived small RNAs (tsRNAs) and tRNA-derived stress-induced small RNAs (tiRNAs), are produced by the activity of endo- and exo-nucleolytic cleavage at different loops of pre-tRNA or mature tRNA [16]. Such processing is very common, as up to 25% of all ncRNA reads can be represented by tRNA fragments [9].

The role of tRFs is very versatile, from the regulation of metabolic processes [17] to the control over cell division and proliferation [18], the control of genome stability [19], and pathogen response [20]. In pathology, tRFs were found to be differentially expressed in various cancers [21] and amyotrophic lateral sclerosis [22]. Various tiRNAs were even proposed to be hallmarks of various diseases [23,24,25] and as therapy options; in the latter, specific tRFs derived from exosomes were shown to alleviate systemic lupus erythematosus [26].

ncRNA fragments were shown to be upregulated in response to various stresses, including arsenite treatment and heat shock [27], nutrition deficiency and hypothermia [28], tissue hypoxia [29], and UV radiation [30]; more information about the role of tiRNAs in stress can be found elsewhere [31]. Angiogenin (ANG) is one of the endonucleases induced by various cellular stresses that cleave tRNAs into tiRNAs [27].

It appears that one of the reasons why tiRNA production is induced by stress is the inhibition of translation; similar to tiRNAs, Val-tRF was shown to be induced by stress and bind to ribosomal units to inhibit translation [32]. Hyperosmosis-induced tiRNAs competitively inhibit the binding of cyt c to the apoptotic protease-activating factor 1 (APAF1) protein, preventing the formation of apoptosome and the activation of cell death [33].

The role of ncRNA fragments in response to stress may also lie in the direct control of the RNA silencing mechanism, either by interference with RNA-induced silencing complex (RISC) formation or by participating in it. In the former, tiRNAs may bind Dicer and prevent it from cleaving double-strand RNAs, as was shown in Drosophila [34]. Being smaller in size than tiRNAs, tRFs may be more ideally suited to be a part of RISC and participate in direct silencing by translational inhibition or cleavage [35].

In our previous work, we performed an extensive comparison of ncRF abundance between males and females and between different brain regions in healthy rats [36]. So, in this work, we have focused on the comparison of control samples with scattered radiation samples. We will emphasize the differences between brain regions or between males and females only in the context of the response to radiation.

## 2. Results

### 2.1. Comparison of ncRNA Reads between Different Brain Regions in Male and Female Rats in Control versus Scatter Radiation Exposure

**Figure 1:**
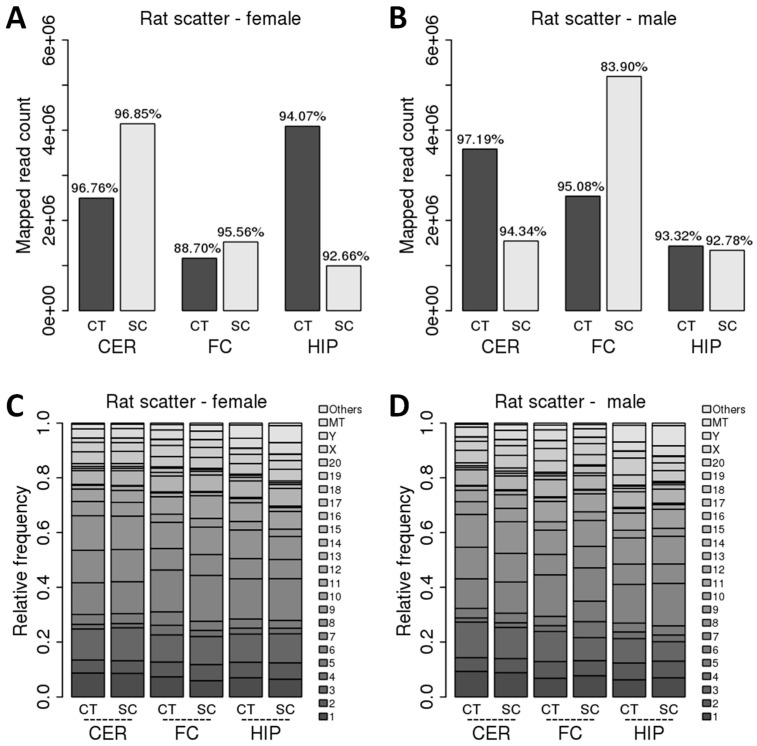
Mapping of reads to various ncRNAs in the FC, HIP, and CER of male and female rats. In (**A**) (female) and (**B**) (male), the *y*-axis shows the number of mapped reads; numbers over the bars show the percentage of mapped reads. In (**C**) (female) and (**D**) (male), the *y*-axis shows the mapping to a specific chromosome out of 1.0, representing 100%. In all cases, the *x*-axis shows the brain regions for the control (Ct) and scatter-irradiate (SC) animals.

Every sequenced sample had at least 1.0 million reads, and the number of reads varied from ~1.0 to ~6.0 million (Figure 1A, *y*-axis). We first analyzed the mapping of reads to the rat genome. The percentage of mapped sequences ranged from 83.9% to 97.2%, with a significant difference between the scattered and control samples for the FC regions of both male and female rats (Figure 1A,B). An analysis of the read distribution by chromosomal position showed a similarity between the control and scattered samples in terms of the CER and HIP of male and female rats, while there were some differences observed in FC (Figure 1C,D).

A further analysis of reads was conducted based on pooled data (from several biological repeats). This was carried out because the processing of some ncRNAs into ncRFs is quite rare, and we wanted to obtain a larger number of ncRFs to characterize. It should be noted that there is very little variation in the quality of reads or the distribution of any specific ncRNA groups among biological repeats; thus, the pooling sequence data from biological repeats did not affect the quality of the analysis.

**Figure 2:**
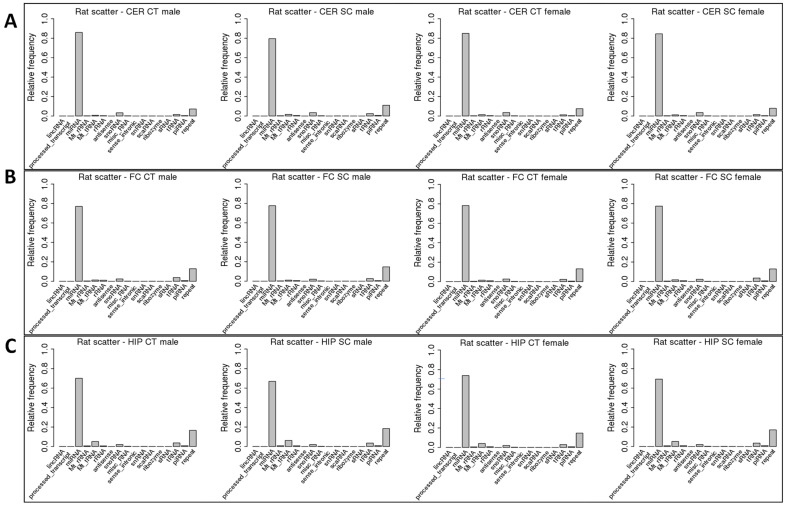
Mapping of sequence reads to various ncRNAs in the cerebellum (CER) (**A**), frontal cortex (FC) (**B**), and hippocampus (HIP) (**C**) of control and scatter-irradiated male and female rats. The *y*-axis shows the mapping out of 1.0, representing 100%, while the *x*-axis shows various types of ncRNAs.

The analysis of the distribution of the size of ncRNA reads showed that they were all ~22 nt in size, and there was no difference between the control and scattered radiation samples (Figure S1). This is likely because reads were predominantly mapped to miRNAs, which are ~22 nt in size, on average (Figure 2).

We next mapped the reads to all known non-coding RNAs. The miRNA fraction was by far the most predominant, making up ~80% of reads mapped to miRNAs. The second largest fraction was repeat-associated ncRNAs (ra-ncRNAs), which made up 10–12% of all reads. Scatter radiation resulted in a decrease in miRNA reads from 85% to 79% in male CER, while ra-ncRNA reads increased from 6% to 10%; no such changes were observed in females (Figure 2A). In HIP, in response to scatter radiation, the male miRNA fraction dropped from 70% to 66%, while the mt-RNA and ra-ncRNA fractions increased. Similarly, in females, the miRNA fraction decreased from 74% to 69%, and the mt-RNA and ra-ncRNA fractions increased (Figure 2C). No changes in male or female rats were observed in FC in response to radiation (Figure 2B).

The GC content of ncRNAs may affect their ability to bind DNA or RNA molecules; RNAi efficiency negatively correlates with GC content [37]. We thus checked the GC content of ncRNAs in response to scatter radiation (Figure S2A). We observed an increase in the GC content of antisense RNAs from 33% to 43% in response to scatter radiation in the FC of females but not males (Figure S2B). Similarly, the GC content increased from 44% to 53% in the CER of females (Figure S2A) and decreased from 57% to 49% in the HIP of females (Figure S2C). In contrast, no changes in GC content were observed in males. Changes in the GC content of antisense RNAs in response to radiation in females’ brain regions may represent a sex-specific stress response.

**Figure 3:**
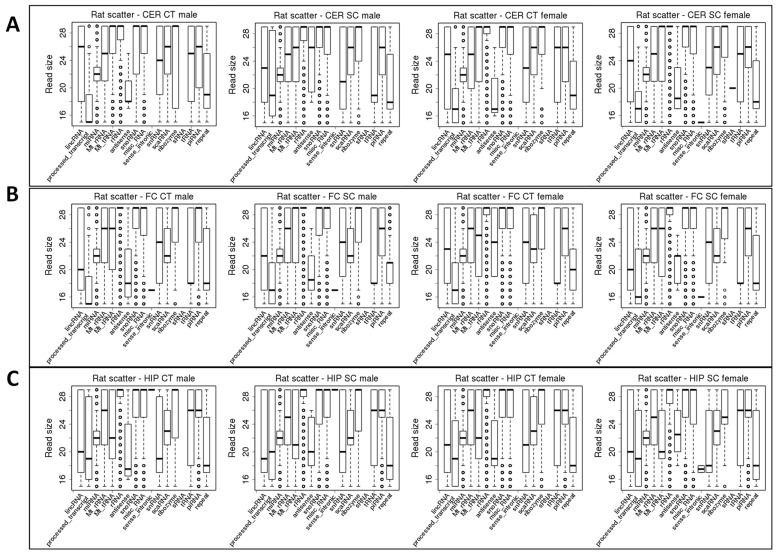
Differences in the size of the reads mapping to different ncRNAs in the FC, HIP, and CER regions of control and scatter radiation-exposed male and female rats. (**A**) Read size in the FC of control and scatter-irradiated males and females; (**B**) Read size in the HIP of control and scatter-irradiated males and females; (**C**) Read size in the CER of control and scatter-irradiated males and females. The *y*-axis shows the size of the reads, while the *x*-axis lists various types of ncRNAs. The bottom and top of the rectangle indicate the first and third quartiles, respectively. The lower and upper ends of the vertical line extending outside the rectangle represent the minimum and maximum, respectively. The thick horizontal line inside the rectangle is the median, and the circle beyond the rectangle displays an outlier.

We next tested the size of the reads mapping to various ncRNAs. In male cerebellum, scatter radiation changed the size of various ncRNAs (Figure 3A). For example, lincRNA changed from 26 nt to 23 nt, processed transcripts changed from 15 to 19 nt, antisense changed from 18 to 26 nt, snRNA changed from 24 to 21 nt, and tRNA changed from 25 to ~18–19 nt. In contrast, there was almost no change in the size of ncRNAs in the female cerebellum, except for the appearance of sense-intronic ncRNAs and sRNAs in response to scatter radiation. In the frontal cortex, no significant changes occurred in response to scatter radiation, except the appearance of sense-intronic ncRNAs in females (Figure 3B). In the male hippocampus, there was an increase in the size of antisense ncRNAs from ~17 to 20 nt (Figure 3C). A similar change was observed in the female hippocampus, where the antisense ncRNA size increased from 18–19 nt to 22–23 nt. In the female hippocampus, scatter radiation also resulted in the appearance of sense-intronic ncRNAs that were ~18 nt in size; this fraction was not present in the control females (Figure 3C).

### 2.2. ncRF Analysis

The processing of ncRNAs to ncRFs often occurs in a biased way, where one of the ends is processed more frequently [31]. Among all ncRNAs, tRNAs, rRNAs, snoRNAs, and snRNAs are the most commonly processed ncRNAs. We previously demonstrated that ncRNAs are processed in the brain in a biased way, with the bias toward the 5′ end for tRNA and ra-ncRNA, as well as toward the 3′ end for snRNA, snoRNA, and rRNA [32]. Here, we show that no changes were observed in the processing in response to scatter radiation as compared to control animals (Figure S3).

ncRNAs can be processed into ncRFs at a different rate, and this processing can be influenced by stress [38]. Thus, in the next step, we analyzed the number of processed ncRF reads. Since there was a different number of reads in each sample, we prorated all ncRF reads to the scatter-irradiated male frontal cortex sample (FC_SR_M).

**Figure 4:**
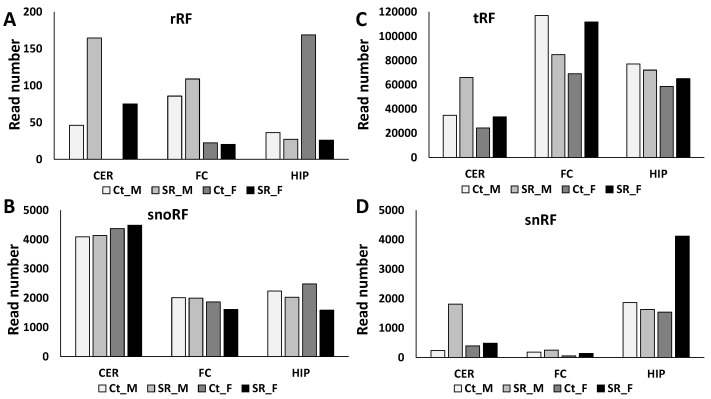
Number of rRF (**A**), snoRF (**B**), tRF (**C**), and snRF (**D**) reads in the CER, FC, and HIP of control and scatter-irradiated male and female rats. “Ct_M”—control male, “SR_M”—scatter radiation-exposed males, “Ct_F”—control females, “SR_F”—scatter radiation-exposed males. The *y*-axis shows the prorated read number, while the *x*-axis shows the group of animals.

We previously reported that tRF reads were the most abundant among the four ncRF read types analyzed, with FC having the most reads and male rats consistently having more reads [36]. Scatter radiation increased the number of reads in the CER of both males and females (Figure 4C). In the FC, the reads in males decreased, while in females, the reads increased in response to scattered radiation. No changes were observed in the HIP.

rRF reads also increased in the CER of males and females in response to scattered radiation, and they slightly increased in the male FC (Figure 4A). In contrast, in the HIP, rRF reads dramatically decreased in females in response to radiation.

snoRF reads did not change in response to scattered radiation, with the exception of the female HIP.

snRF reads increased in the male CER and the male HIP in response to radiation.

We then checked the abundance of reads mapping to either the 5′ or 3′ end. The tRF and snRF reads contained only 5′ end reads, while rRF and snoRF contained both (Figure S4). In the male CER, the control animals had only 5′ end reads, while the scatter-irradiated animals had only 3′ end reads (Figure S4A,B). In the FC, the control males did not have 3′ end rRF reads, while the irradiated males had a large fraction. Finally, in the HIP, both the male and female control animals had 3′ end reads, while in the scatter-irradiated animals, these reads disappeared. snoRFs had a larger fraction of 3′ end reads (Figure S4C,D). While the total fraction of snoRF reads did not change in response to radiation in the CER, the 5′ end fraction decreased substantially in males (Figure S4C).

**Figure 5:**
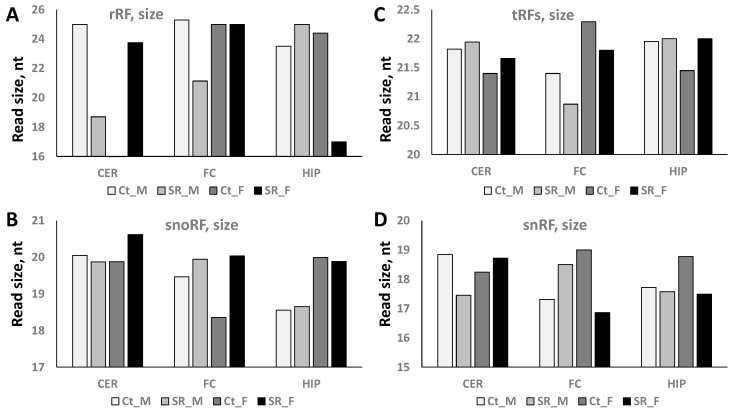
Size of rRF (**A**), snoRF (**B**), tRF (**C**), and snRF (**D**) reads in the CER, FC, and HIP of control and scatter-irradiated male and female rats. “Ct_M”—control male, “SR_M”—scatter radiation-exposed males, “Ct_F”—control females, “SR_F”—scatter radiation-exposed males. The *y*-axis shows the size of the reads, while the *x*-axis shows the group of animals.

ncRNAs are also often processed into reads of different sizes; thus, we checked the size of the ncRF reads. In tRFs, we noticed an increase in the average read size in the CER of both animals, a decrease in both animals in the FC, and an increase in the female HIP in response to radiation (Figure 5C). In males, the rRFs read size is drastically decreased in the CER and FC, while in females, it drastically decreased in the HIP in response to radiation (Figure 5A). The snoRF read size increased in the CER and FC of females in response to scatter radiation (Figure 5B). Finally, the snRF read size decreased in the CER of males and increased in the FC of males, while decreasing in the FC and HIP of females in response to radiation (Figure 5D). Figure S5 provides the details of the changes in read size in the 5′ and 3′ fractions.

### 2.3. Correlation between ncRNA Reads and ncRF Reads

**Figure 6:**
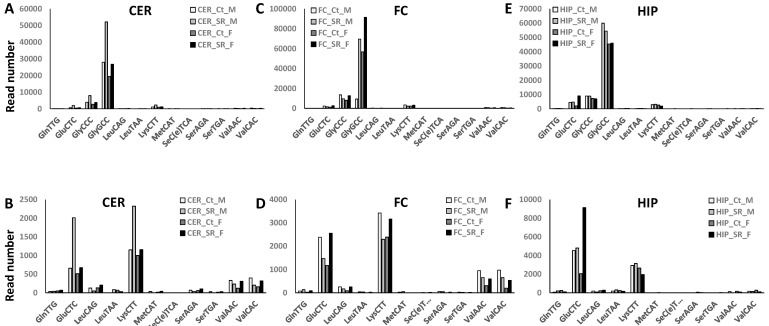
Comparison of the number of tRF reads mapping to different tRNAs in control and scatter radiation-exposed groups of males and females in the CER (**A**), FC (**C**), and HIP (**E**) brain regions. Since reads mapping to Gly were predominant, we generated another set of figures omitting Gly reads—CER (**B**), FC (**D**), and HIP (**F**). The *y*-axis shows the number of reads mapping to tRNA. The *x*-axis shows the tRNA to which the reads mapped by type.

ncRNAs are processed into ncRFs at a different rate; while some are used more frequently, others may not be processed at all. We recently showed that the tRF-Gly reads were the most predominant, representing ~90% of all tRF reads [32]; we also reported that there was no significant difference between different brain regions in males or females in terms of the number of those reads. Here, we show that scatter radiation exposure increased the number of GlyGCC and GlyCCC mapping reads in the CER of both males and females (Figure 6A). An analysis of less abundant reads in the CER showed that reads mapping to GluCTC and LysCTT also increased in number in both sexes, more prominently so in males; at the same time, reads mapping to LeuCAG, ValAAC, and ValCAC decreased in males and increased in females (Figure 6B). In FC, an increase in GlyCCC but not in GlyGCC was observed in both sexes in response to radiation (Figure 6C). In the less abundant group, in the FC, reads mapping to GluCTC, LeuCAG, LysCTT, ValAAC, and ValCAC decreased in males and increased in females in response to radiation (Figure 6D). Changes in HIP were less drastic—there was a decrease in GlyGCC reads in males, an increase in GluCTC in females, and a decrease in LysCTT in females. Thus, it appears that there is a sex-specific response to radiation in all three brain regions.

**Figure 7:**
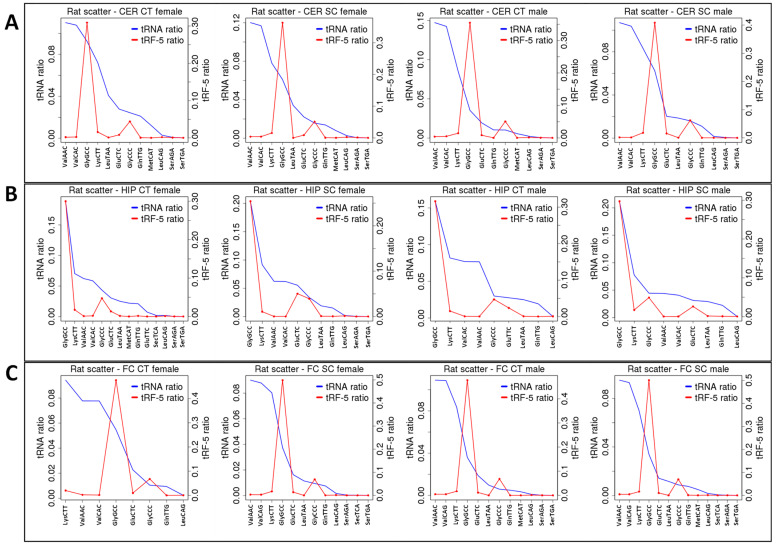
The enrichment of tRFs processed from tRNAs in the CER (**A**), HIP (**B**), and FC (**C**) of control and SC animals. “HIP_Male”—hippocampus of male rats; “HIP_Female”—hippocampus of female rats; “FC_Male”—frontal cortex of male rats; “FC_Female”—frontal cortex of female rats; “CER_Male”—cerebellum of male rats; “CER_Female”—cerebellum of female rats; “Ct”—control; “SC”—scatter radiation. The *y*-axis shows specific tRNA and tRF-5′. The *x*-axis shows specific classes of tRNAs. When the tRF peak is larger than the tRNA peak, there is an enrichment, while when it is lower, there is underrepresentation.

To find out whether all tRNAs were processed into tRFs at the same frequency, we have plotted the number of tRNA and tRF reads on the same graph (Figure 7). In the CER, tRF-GlyGCC and tRF-GlyCCC were enriched in all samples, and in response to radiation, they increased in females and slightly decreased in males (Figure 7A). In the HIP, tRF-GlyGCC was enriched but not affected by radiation, while GlyCCC increased in females but not in males (Figure 7B). In the FC, tRF-GlyGCC and tRF-GlyCCC were enriched, but in response to radiation, only tRF-GlyGCC decreased in females but not in males (Figure 7C).

Thus, it appears that certain tRNAs are more frequently processed to tRFs, and their processing is altered in response to radiation in a brain- and sex-specific manner.

**Figure 8:**
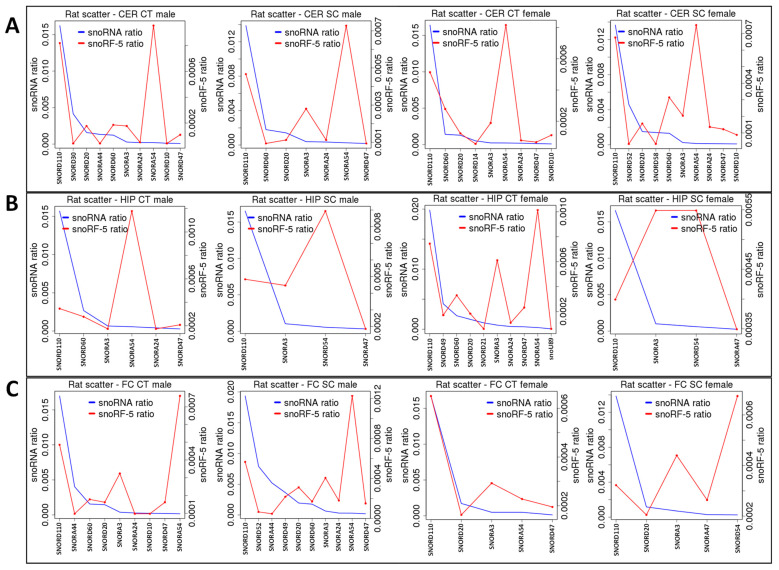
snoRF-5′ enrichment from snoRNAs in the CER (**A**), HIP (**B**), and FC (**C**) of control and SC animals. “HIP_Male”—hippocampus of male rats; “HIP_Female”—hippocampus of female rats; “FC_Male”—frontal cortex of male rats; “FC_Female”—frontal cortex of female rats; “CER_Male”—cerebellum of male rats; “CER_Female”—cerebellum of female rats; “Ct”—control; “SC”—scatter radiation. The *y*-axis shows specific snoRNA and snoRF-3 ratios. The *x*-axis shows specific snoRNA/snoRF. When the snoRF peak is larger than the snoRNA peak, there is an enrichment, while when it is lower, there is underrepresentation.

A previous analysis of the processing of snoRNAs into snoRFs showed enrichment in several types of snoRFs, including snoRA54, snoRA3, snoRA60, and snoRD20 [36]. Radiation exposure changed this enrichment in several cases. Specifically, in the group of snoRFs stemming from the 5′ end, in the male CER, snoRD20 and snoRA60 lost enrichment, while in the female CER, snoRA24 and snoRD47 became more drastically enriched in response to radiation (Figure 8A). In the HIP, snoRA3-5′ became enriched in both sexes, with males having a larger increase (Figure 8B). In the FC, snoRD20-5′ and snoRA24-5′ were enriched in males, while snoRD10-5′m lost enrichment and snoRA3-5′ and snoRA54-5′ dramatically increased their enrichment in response to radiation (Figure 8C).

**Figure 9:**
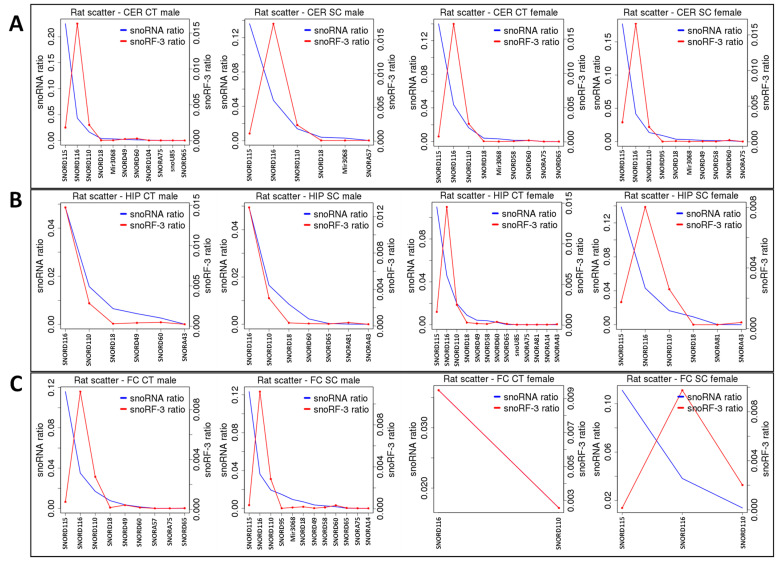
snoRF-3′ enrichment from snoRNAs in the CER (**A**), HIP (**B**), and FC (**C**) of control and SC animals. “HIP_Male”—hippocampus of male rats; “HIP_Female”—hippocampus of female rats; “FC_Male”—frontal cortex of male rats; “FC_Female”—frontal cortex of female rats; “CER_Male”—cerebellum of male rats; “CER_Female”—cerebellum of female rats; “Ct”—control; “SC”—scatter radiation. The *y*-axis shows specific snoRNA and snoRF-3 ratios. The *x*-axis shows specific snoRNA/snoRF. When the snoRF peak is larger than the snoRNA peak, there is an enrichment, while when it is lower, there is underrepresentation.

An analysis of snoRFs stemming from the 3′ end showed no changes in enrichment in the CER (Figure 9A), while in the HIP, in males, snoRA81 became enriched, and in females, snoRD110 became enriched (Figure 9B). At the same time, in the FC, no changes were found in males, while in females, the snoRNA numbers were beyond the threshold (five reads), and it was thus impossible to judge whether changes in the processing had occurred (Figure 9C).

An analysis of the processing of rRNA showed that, in the male FC, scatter radiation strongly increased the enrichment of rRF-5′ (Figure S6).

Finally, plotting snRNAs against snRFs showed that the enrichment of snRF-U1-3′ was increased in all regions in both sexes, with the most dramatic increase in response to radiation observed in the CER of males (Figure S6). A small increase in the enrichment of snRF-U5-3′ was also observed in the male FC exposed to scatter radiation.

### 2.4. Prediction of Targets of ncRFs in Response to Radiation Using miRDB

**Figure 10:**
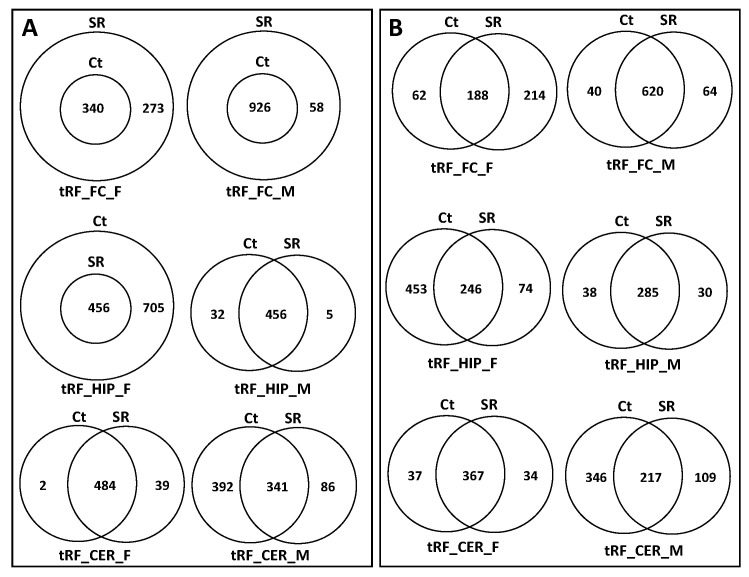
Venn diagrams of overlapping target genes: (**A**) as analyzed by miRDB and target pathways; (**B**) as analyzed by DAVID of tRFs in brain regions of control and scatter-irradiated male and female rats.

To predict potential targets of ncRFs, we used the miRNA target prediction database (miRDB) (Supplementary File S1). For tRF targets, we found that, in the FC, in response to radiation, there was a substantial increase in the potential number of targets, with changes in females being more dramatic. The predicted target genes in the Ct group fully overlapped with those of the SR group (Figure 10A; Supplementary File S2). In tRF in the HIP, there was a reduction in the number of targets in both female and male animals, which was much larger in females. The Stac, Lpin2, Esco1, Nr6a1, and Zfp53 genes were unique targets of tRF in the HIP in males.

In the CER, the number of tRF targets increased in females and decreased in males in response to radiation; in males, there was a large number of targets that were unique in Ct and irradiated samples (Figure 10A).

**Figure 11:**
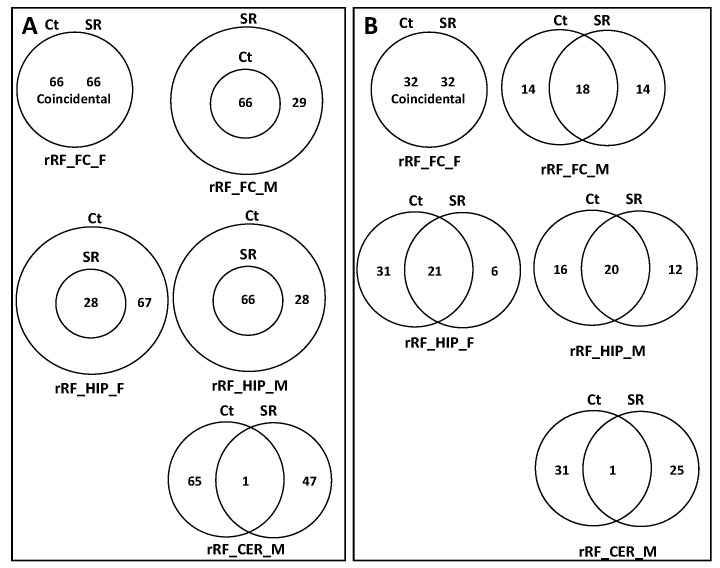
Venn diagrams of overlapping target genes: (**A**) as analyzed by miRDB and target pathways; (**B**) as analyzed by DAVID of rRFs in brain regions of control and scatter-irradiated male and female rats.

We next analyzed the targets of rRFs (Supplementary Files S1 and S2; Figure 11A). In the FC, radiation did not change the targets, while in males, it increased, and all targets in the control overlapped with the irradiated samples. In the HIP, there was a reduction in the number of targets in both sexes, more so in females. In the CER, radiation led to drastic changes in males, with only a single gene overlapping between the Ct and SR samples. No targets were found in females, as there were very few rRFs to work with.

**Figure 12:**
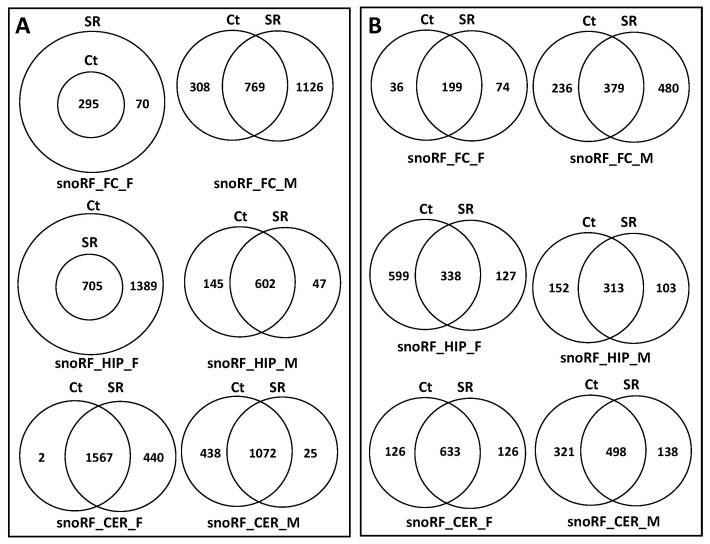
Venn diagrams of overlapping target genes: (**A**) as analyzed by miRDB and target pathways; (**B**) as analyzed by DAVID of snoRFs in brain regions of control and scatter-irradiated male and female rats.

An analysis of unique snoRFs targets showed an increase in the FC in both sexes, with more drastic changes in males (Figure 12A; Supplementary Files S1 and S2). In the HIP, there was a decrease in both sexes, which was more extreme in females in response to radiation. In the CER, there was an increase in females but a decrease in males.

**Figure 13:**
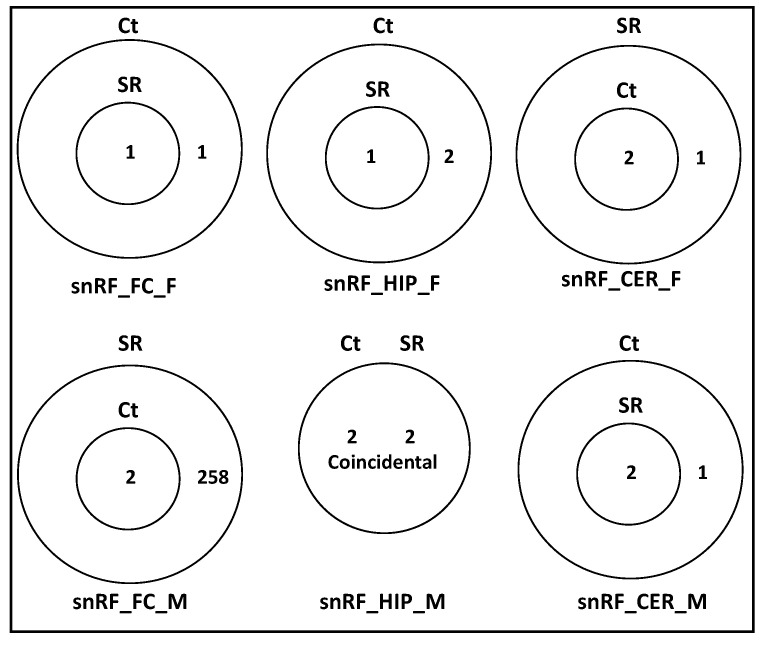
Venn diagrams of overlapping target genes (analyzed by miRDB) of snRFs in brain regions of control and scatter-irradiated male and female rats.

There was a small number of snRF unique targets in all samples, except for the FC in males, where there was a massive increase in the number of targets (Figure 13; Supplementary Files S1 and S2).

### 2.5. Analysis of Overlapping and Unique Pathways targeted by ncRFs Using DAVID

When we performed the analysis of unique and overlapping pathways, we found that, for tRFs, the number of targeted pathways increased in the FC and decreased in the HIP and CER in both sexes (Figure 10B; Supplementary File S3). For rRFs, no changes were observed in the female FC, while radiation resulted in the appearance of unique pathways for both the Ct and SR groups. Additionally, for rRFs, radiation resulted in a decrease in the number of target pathways in the HIP in both sexes and in the CER in males (Figure 11B; Supplementary File S3). For snoRFs, the number of targeted pathways increased in the FC and decreased in the HIP in both sexes and in the CER in males (Figure 12B). No pathways could be found in snRFs due to the low number of snRF targets.

**Figure 14:**
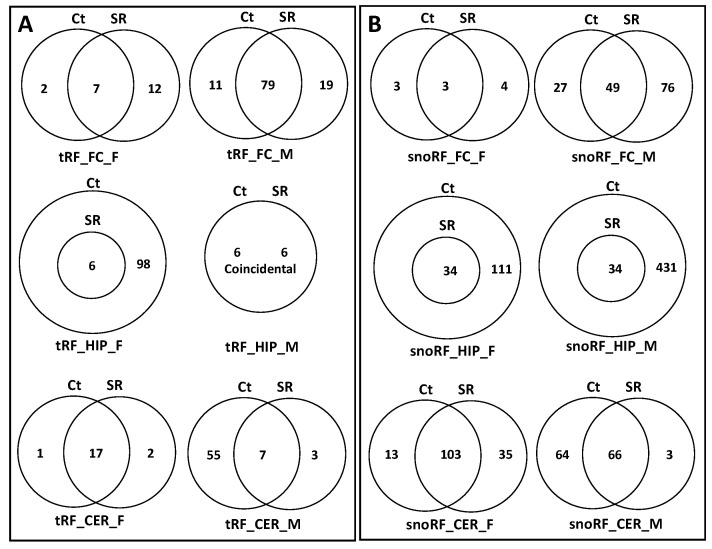
Venn diagrams of overlapping target genes (analyzed by miRDB) of tRFs (**A**) and snoRFs (**B**) in brain regions of control and scatter-irradiated male and female rats after Benjamini correction.

We next performed the analysis of significantly enriched pathways by performing a Benjamini correction. There were no significantly enriched pathways for the rRF and snRF targets. For tRF and snoRF, for the sake of space, we will only mention pathways related to brain and neuronal activity. For tRF, in the FC_F group, the control had two unique pathways—the presynaptic membrane and synapse—while the SR group had seven unique pathways, among which there were the glutamatergic synapse, cAMP signaling pathway, cell junction, and several others (Figure 14A; Supplementary File S4). In males, the FC group had many unique, significantly enriched pathways; specifically, the Ct group had glutamatergic synapse pathways, while the SR group had brain development and neuronal cell body pathways.

In the HIP_F control group—axon guidance, glutamatergic synapse, neuron projection, postsynaptic density, and neurotrophin signaling; no difference was observed in HIP_M.

In the CER_M SR group, there was learning; in the Ct group, there were neurotrophin signaling, axon guidance, dendritic spine, brain development, and neuron projection. In CER_F, only a few pathways were altered, and none were specific to brain/neuron function.

In the targets of snoRFs, the significantly enriched pathways in the FC_F SR group were the positive regulation of neuron projection development, growth cone, and axon guidance; in the FC_M control, there were neuron projection, synapse, and postsynaptic membrane, while in the SR group, there were dendrite cytoplasm, axonogenesis, glutamatergic synapse, and dopaminergic synapse.

In the HIP, radiation resulted in a significant decrease in the number of pathways targeted by snoRFs, more prominently in males. The control HIP_F group had the following pathways significantly enriched: dendrite morphogenesis, axon guidance, axonogenesis, and neurogenesis. The control HIP_M group had dendrite cytoplasm, axonogenesis, axon guidance, neuron differentiation, and many others (Table 1). In CER_F, the SR group had the following uniquely enriched pathways: axonogenesis and dendrite. In the CER_M Ct group, there were the regulation of neuron projection development, axonal growth cone, amphetamine addiction, and the positive regulation of neuron projection development.

## 3. Discussion

We previously showed tissue- and sex-specific differences in the expression of ncRNAs and their processing to ncRFs. Here, we show that scatter radiation also results in sex- and tissue-specific changes in the expression and processing of ncRFs, as well as changes in the enrichment of predicted targets of processed ncRFs. Table 2 shows a detailed summary of the main findings of this study (Table 2).

Scatter radiation is known to cause radiation-induced bystander effect (RIBE)—various physiological and molecular changes in the tissues not directly exposed to radiation [39]. The irradiation of internal organs such as the liver results in changes in gene expression in the brain as well as changes in animal behavior [40]. Moreover, RIBEs are often sexually dimorphic [41], with the effects being more pronounced in females [42].

The mechanism of RIBE is not clear, but it appears that exosomes and extracellular vesicles (EV) play a critical role in it, as they carry various coding and non-coding RNAs, including miRNAs, rRNAs, snRNAs, and lncRNAs [43,44].

### 3.1. Brain- and Sex-Specific Differences in ncRNAs in Response to Scatter Radiation

Stress—specifically, radiation—causes massive changes in the expression of short and long non-coding RNAs [45]. Previous studies demonstrated sex-dimorphic changes in miRNA expression in the mouse brain in response to radiation [46] or in animals with stroke [47].

In our work, we observed decreased miRNA expression and the increased expression of ra-ncRNA reads in the HIP and CER, as well as an increased number of mtRNA-associated reads in the HIP. Changes in miRNA and ra-ncRNA expression in the CER were sex-specific—observed in males but not in females.

An increase in the number of ra-ncRNAs in response to scatter radiation may be pathological. Ra-ncRNAs can bind and sequester RBPs, including those that regulate alternative splicing, and thus can decrease the number of protein variants available to the cell [48]. In addition, it appears that these ncRNAs can be translated in a non-AUG-dependent manner and produce toxic short peptides that are potentially harmful to the cell [49]. It can be further speculated that the accumulation of ra-ncRNAs is part of the so-called radio- and/or chemo-brain—a cognitive decline observed in patients treated for cancer [50].

We observed changes in the GC content and the read size of antisense ncRNAs. The antisense ncRNAs are predominantly from long-ncRNAs (lncRNAs), which stem from the antisense strands of coding genes. LncRNAs are known to predominantly regulate the expression of their own genes, mainly acting at the transcription level, although some lncRNAs also control RNA stability by acting as miRNA sponges and can favor translation by recruiting sense RNAs to polysomes or control splicing by interfering with spliceosomes [51]. The expression of lncRNAs in the brain is sex-specific [10]. Our previous work also showed that the GC content of antisense ncRNAs was significantly lower in all brain regions of females than in those of males. It is known that a higher GC content is closely associated with the formation of the RNA secondary structure, which correlates with biological function that is not related to encoding proteins [52]. Thus, decreased GC content in the pool of differentially expressed lncRNAs may suggest an increased function of lncRNA in female brains in the regulation of the expression of other genes. Our current work shows that there is not only a sex-specific difference in the endogenous processing of antisense ncRNAs in the brain but also a change in GC content in response to scatter radiation in females. It is still not quite clear what the mechanism of such differences is and what the biological meaning of it would be. Since GC content affects splicing [53], and differences in splicing are sex- and tissue-specific in physiological and pathological conditions [54], it is possible that changes in GC in the pool of antisense lncRNAs in females represent a sex-specific response to scatter radiation.

We also observed the appearance of sense-intronic ncRNAs in response to scatter radiation; the effect was also brain- and sex-specific. There exists very little research on this type of ncRNAs in the literature, but it was shown that they can be induced in response to the androgen hormone and retinoic acid [55], as well as differentially regulated in several cancers (reviewed in [56]). Our report is likely the first one demonstrating the differential expression of sense-intronic ncRNAs in response to scatter radiation.

### 3.2. Difference in the Processing of ncRFs in Response to Scatter Radiation

We observed changes in the ncRF read number and size in response to radiation and changes in the distribution of rRF reads—the appearance and disappearance of rRF-3′ reads in various brain regions. As ncRFs have a variety of functions in controlling gene expression and they are commonly present in EVs, it is not surprising that they are altered in response to scatter radiation.

tRFs and tiRNAs were found to be present in exosomes [57]. In fact, small EVs contain up to 50% tRNA fragments from the entire pool of small RNAs; the total content and diversity of tiRNAs and fragments in EVs appear to be dependent on the origin of those EVs—those stemming from adipose tissues had over 50% tRFs, while those from bone marrow had only 23–25% [58].

In the HIP, changes in the size and abundance of ncRFs were only observed in females, while in the CER/FC, they were observed in both sexes. Changes in tRF abundance in response to radiation were mainly observed in the CER and FC, and they were more or less similar in males and females, while in the HIP, there were fewer changes, and they were sex-specific.

Our previous work [36] and the work of others [59] demonstrated that there is a different rate of processing of tRNAs—some are more frequently cleaved into tiRNAs and tRFs than others. In this work, we show that tRF-GlyGCC and tRF-GlyCCC are the most frequently processed and exhibit tissue- and sex-specific changes in expression. Changes in the expression of these two tRFs were more pronounced in females compared to males and were more drastic in the CER. Changes in other snoRFs in the HIP were minor, while in the CER and FC, they were more drastic and sex-specific. In the CER and FC, snoRF-5’ more pronounced in females, and in FC, snoRF-3’ were also processed more efficiently in females.

Many tiRNAs and tRFs are differentially expressed in response to stress, with most data coming from the realm of cancer biology [60]. tiRNAs and tRFs are also found to be differentially expressed in other diseases/conditions, including diabetic retinopathy [61], endometriosis [25], spinal cord injury [62], and many more. Some are involved in multiple pathologies—5′tiRNA-His-GTG was shown to respond to tissue hypoxia and may be involved in malignancy [29].

A previous report showed that the ultraviolet irradiation of skin results in the differential expression of several tsRNAs, upregulating tRF-Val-AAC-012, tRF-Pro-AGG-012, tRF-Val-CAC-018, and tRF-Val-AAC-031 and downregulating tRF-Arg-CCT-002, tRF-Trp-TCA-001, tiRNA-Ser-GCT-001, tRF-Gly-CCC-019, tRF-Ala-TGC-001, and tRF-Ala-TGC-002 [30]. It was speculated that tRF-Gly-CCC-019 targets the ras-related C3 botulinum toxin substrate 1 (Rac1) gene in the WNT signaling pathway and is one of the important players in acute UVB-induced skin injury [30].

### 3.3. Predicted Targets

RIBE-induced exosomes can play positive and negative roles in the cells—ncRNAs and ncRFs in exosomes and EVs can regulate gene expression at the destination cells by either activating defense mechanisms or causing instability, increasing inflammation and preparing cells for malignization events [63].

We used miRDB software to call potential targets of differentially expressed ncRNAs. We found that there was a decrease in the expression of tRF in the HIP targeting a set of genes, including the Stac, Lpin2, Esco1, Nr6a1, and Zfp53 genes; this would likely lead to the increased expression of these genes.

Stac is encoded by a developmentally regulated brain transcript; it was found to suppress the Ca^2+^-dependent inactivation of neuronal l-type Ca^2+^ channels [64]. Lpin2 encodes Lipin-2, a protein likely involved in lipid metabolism, and has been found to be polymorphic in autosomal disease myopia [65] and Majeed syndrome, a human inflammatory disorder [66]. NR6A1, nuclear receptor subfamily 6, group A, member 1, regulates lipid metabolism through the mammalian target of rapamycin complex 1 [67]. Hippocampal NR6A1 impairs CREB-BDNF signaling and leads to the development of depression-like behaviors in mice [68]. Esco1 belongs to a conserved family of acetyltransferases involved in sister chromatid cohesion. Esco1 is upregulated in military personnel with PTSD [69]. Zfp53 encodes zinc finger protein 53, a transcription factor with an unknown function. Thus, it is possible that the increased expression of these genes due to the lower level of tRFs targeting them may contribute to the impairment of CREB-BDNF signaling and even PTSD-like symptoms in response to scatter radiation. Of course, the expression of these genes needs to be tested, and their role in the aforementioned processes needs to be confirmed.

An analysis of annotated targets using DAVID revealed a large number of pathways involved in neuronal function, including axon guidance, axonogenesis, chemical synaptic transmission, dendrite cytoplasm, excitatory postsynaptic potential, glutamatergic synapse, neuron differentiation, the positive regulation of dendrite development, postsynaptic cell membrane, postsynaptic density, the regulation of neurotransmitter secretion, the regulation of synaptic transmission, retrograde endocannabinoid signaling, response to cocaine, cocaine addiction, and morphine addiction. Most of these pathways were underrepresented in scatter radiation samples in the HIP and CER, and several of them were overrepresented in FC, possibly suggesting that scatter radiation alters the normal function of all these pathways maintaining normal brain function, and this response is brain-region specific. It is very much possible that changes in the expression of genes involved in these pathways in response to scatter radiation contribute to “radio-brain”, thus suggesting the essential role of tRFs and snoRFs in this pathology.

## 4. Materials and Methods

### 4.1. Animal Model Used in This Work

The tissues from animals used in this experiment were previously used in the experiment that aimed to establish radiation effects on the brain [41]. The total RNA was extracted from different brain regions of control and sham-irradiated 3-month-old male and female Long Evans rats purchased from Charles River; three animals of each sex were used in the analysis. All animals were housed in a pathogen-free controlled facility with a light/dark cycle of 12/12 h. Food and water were given ad libitum. The handling and care of the animals were conducted in accordance with the recommendations of the Canadian Council for Animal Care and Use. All procedures were approved by the University of Lethbridge Animal Welfare Committee.

### 4.2. Sequencing

The sequencing of the ncRNA was carried out with the Genome Analyzer IIx. We used single-sequence reads of 36 nt; these reads include a 7 nt adaptor and a 29 nt sequence of ncRNA. The sequencing data were analyzed by pooling biological repeats together.

The sequencing reads were processed from the fastq format and then aligned to the known ncRNA sequences using Bowtie with the option of “-v 2-best”. To identify a specific type of ncRNA read in different tissues, all ncRNA reads were classified and presented as a fraction of 1 for their average frequency of occurrence.

### 4.3. The Identification and Description of ncRFs

ncRNA fragments (ncRFs) were defined as reads mapping to the 5′-end and 3′-end of the ncRNAs. Since the maximum length of ncRNA reads in our samples would be 29 nt, we limited the analysis of reads to ≤27. Reads of ≥29 nt could potentially be from longer sequences. The fraction of ≤27 nt is likely the processed fragments of ncRNAs. Only those ncRNAs that had ≥5 reads mapping to them were included in the analysis.

### 4.4. The Analysis of the Distribution of ncRF Reads across the Entire Length of Precursor ncRNAs

Each ncRNA sequence, regardless of length, was divided into 10 equal-sized bins, and ncRF reads mapping to each bin were counted across all ncRNAs in each ncRNA type. The distribution of ncRF reads was calculated by dividing the read count in each bin by the total read count in all bins in each ncRNA type.

### 4.5. The Analysis of the Enrichment of ncRFs Relative to the Number of ncRNA Precursors

ncRNAs and ncRFs were extracted for each ncRNA type. The enrichment was calculated by dividing the number of ncRF reads (those that were ≤27 nt) by the number of ncRNA reads (those that were ≥29 nt). The ratio of 1 indicates that there was one ncRF for each ncRNA.

### 4.6. ncRFs Target Prediction Using miRDB

miRDB is an online database tool (www.mirdb.org, accessed on 1 February 2019) used for the prediction of miRNA targets. We submitted the ncRF sequences into the miRDB web interface, and miRDB returned the predicted targets. The unique ncRF sequences were uploaded to miRDB, and the predicted target genes were retrieved along with their target scores. The scores greater than 80 are most likely to be real [70]. Therefore, the target genes of the unique ncRF sequences with the prediction target score greater than 80 were selected from the miRDB-predicted targets. Each miRDB excel file (Supplementary File S2) consists of three worksheets of unique ncRF sequences, targets predicted by miRDB, and unique targets. Venn diagrams for overlapping gene targets were built on the lists of unique targets using the R package VennDiagram (Vienna, Austria).

### 4.7. ncRFs Target Pathway Prediction Using DAVID

The functional annotation of the unique targets predicted by miRDB (Supplementary File S2) was performed using DAVID. The target gene symbols were provided to the DAVID web interface, and the functional annotation charts were generated. The chart displays the functional annotation of the target genes, including significant ontologies and pathways. Venn diagrams were built using groups of statistically significantly different pathways in each category. The cutoff was made at a Benjamini correction of less than 0.05.

## 5. Conclusions

The effect of scatter radiation on the brain is understudied. Previous research demonstrated significant changes in the gene expression in the brain and the behavior of animals exposed to scatter radiation (where only the liver was exposed). These changes were brain- and sex-specific, with more dramatic changes observed in females. In this work, we tested the hypothesis that ncRNA fragments, ncRFs, are differentially expressed in response to scatter radiation. We indeed found many ncRNAs and ncRFs that were differentially expressed, with multiple brain- and sex-specific changes. Although it is hard to estimate the cumulative effect of changes in ncRFs, we can hypothesize that the observed changes would lead to more profound effects in one of the sexes and in a specific brain region.

To further uncover the role of ncRFs, it would be important to analyze their contribution to gene expression using various molecular assays or even using isolated tRFs in well-defined animal studies. It would also be interesting to analyze the exosomes and EVs for the presence of these ncRFs in scatter-irradiated animals. This will allow us to deduce the biogenesis of differentially expressed ncRFs—from the site of irradiation (liver) or in the brain tissues. We suspect it is likely both.

## Figures and Tables

**Table 1:** Summary of uniquely significantly enriched pathways by tRF and snoRF.

Brain Region	tRF		snoRF	
Sex	Ct	SR	Ct	SR
HIP_M			axon guidance, axon, axonogenesis, chemical synaptic transmission, cocaine addiction, dendrite cytoplasm, excitatory postsynaptic potential, glutamatergic synapse, morphine addiction, neuron differentiation, positive regulation of dendrite development, postsynaptic cell membrane, postsynaptic density, regulation of neuro-transmitter secretion, regulation of synaptic transmission, response to cocaine, retrograde endocannabinoid signaling	
HIP_F	axon guidance, glutamatergic synapse, neuron projection, neurotrophin signaling, postsynaptic density		axonogenesis, axon guidance, dendrite morphogenesis, neurogenesis	
CER_M	axon guidance, brain development, dendritic spine, neuron projection, neurotrophin signaling	learning	amphetamine addiction, axonal growth cone, regulation of neuron projection development, positive regulation of neuron projection	
CER_F				axonogenesis, dendrite
FC_M	glutamatergic synapse	brain development, neuronal cell body	neuron projection, postsynaptic membrane, synapse	axonogenesis, dendrite cytoplasm, dopaminergic synapse, glutamatergic synapse
FC_F	presynaptic membrane, synapse	cAMP signaling pathway, cell junction, glutamatergic synapse		axon guidance, growth cone, positive regulation of neuron projection development

**Table 2:** Summary of main findings.

Brain Region	HIP				CER				FC			
SEX	Male		Female		Male		Female		Male		Female	
Ct/SC	Ct	SC	Ct	SC	Ct	SC	Ct	SC	Ct	SC	Ct	SC
Distribution of ncRNA reads												
miRNA	70%	66%	74%	69%	85%	79%						
ra-ncRNA reads	16%	19%	15%	18%	6%	10%						
mtRNA reads	3%	5%	3%	6%								
GC, antisense			57%	49%			44%	53%			33%	43%
ncRNA read size, nt												
lncRNA					26	23						
Processed					15	19						
antisense	17	20	18–19	22–23	18	26						
Sense-intronic				18		X						X
sRNAs						X						
ncRF read number												
tRF reads		↓		↑		↑		↑				
rRF reads				↓		↑		↑		↑		
snoRF reads				↓								
snRF reads		↑		↑		↑						
rRF5′					X							
rRF3′	X		X			X				X		
ncRF read size												
tRF read size				↑		↑		↑		↓		↓
rRF read size				↓		↓				↓		
snoRF read size								↑				↑
snRF read size				↓		↓				↑		↓
tRF abundance												
tRF-GlyCCC						↑		↑		↑		↑
tRF-GlyGCC		↓				↑		↑				
tRF-GluCTC				↑		↑↑		↑		↓		↑
tRF-LysCTT				↓		↑↑		↑		↓		↑
tRF-LeuCAG						↓		↑		↓		↑
tRF-ValAAC						↓		↑		↓		↑
tRF-ValCAC						↓		↑		↓		↑
ncRF processing												
tRF-GlyGCC		=		=		−		++		=		↓
tRF-GlyCCC		=		++		−		++		=		=
snoRD20-5′						−				+		
snoRD60-5′						−						
snoRA24-5′								+		+		
snoRD10-5′								+		−		
snoRD47-5′								+				
snoRA3-5′										++		
snoRA54-5′										++		
snoRA81-3′		+										
snoRD110-3′				+								
rRF-5′										++		
snRF-U1-3′		+		+		++		+		+		+
snRF-U5-3′										+		

“=”—no change or no difference between scattered radiation and control mice; “+”and “++” — enrichment and strong enrichment in ncRF processing, respectively; “−”—underrepresentation of ncRFs; ↑ and ↓ show an increase and decrease, respectively, in read number, size, or abundance, while X shows the presence of a certain class of ncRNA of fragments.

## Data Availability

All data are either presented in the manuscript or provided in the Supplementary Materials.

## Supplementary Materials

The following supporting information can be downloaded at: https://www.mdpi.com/article/10.3390/epigenomes6040035/s1, Figure S1: ncRNA read size distribution; Figure S2: GC content in reads mapping to different ncRNAs in the CER (A), FC (B), and HIP (C) of control and scatter-irradiated male and female rats; Figure S3: Analysis of the reads distribution across the whole length of ncRNAs; Figure S4. Number of rRF and snoRF reads mapping to the 5′ or 3′ ends of rRNA or snoRNAs; Figure S5: Size of the rRF and snoRF reads mapping to the 5′ or 3′ ends of rRNA or snoRNAs; Figure S6: rRF and snRF enrichment from rRNA and snRNA in various brain regions; File S1: List of all miRDB-predicted targets; File S2: Unique and common targets of various ncRFs; File S3: List of GO terms (pathways) predicted by DAVID; File S4: List of GO terms (pathways) predicted by DAVID after Benjamini correction.
