# Supplementary material for: Scatter Irradiation of Rat Brain Triggers Sex- and Brain Region-Specific Changes in the Expression of Non-Coding RNA Fragments
Source: Epigenomes. 2022 Oct 12;6(4):35. doi: 10.3390/epigenomes6040035 (PMC9624364; doi:10.3390/epigenomes6040035)
Supplement: Supplementary file 1 [file epigenomes-06-00035-s001.zip › epigenomes-1922357-Supplementary figures.pdf]

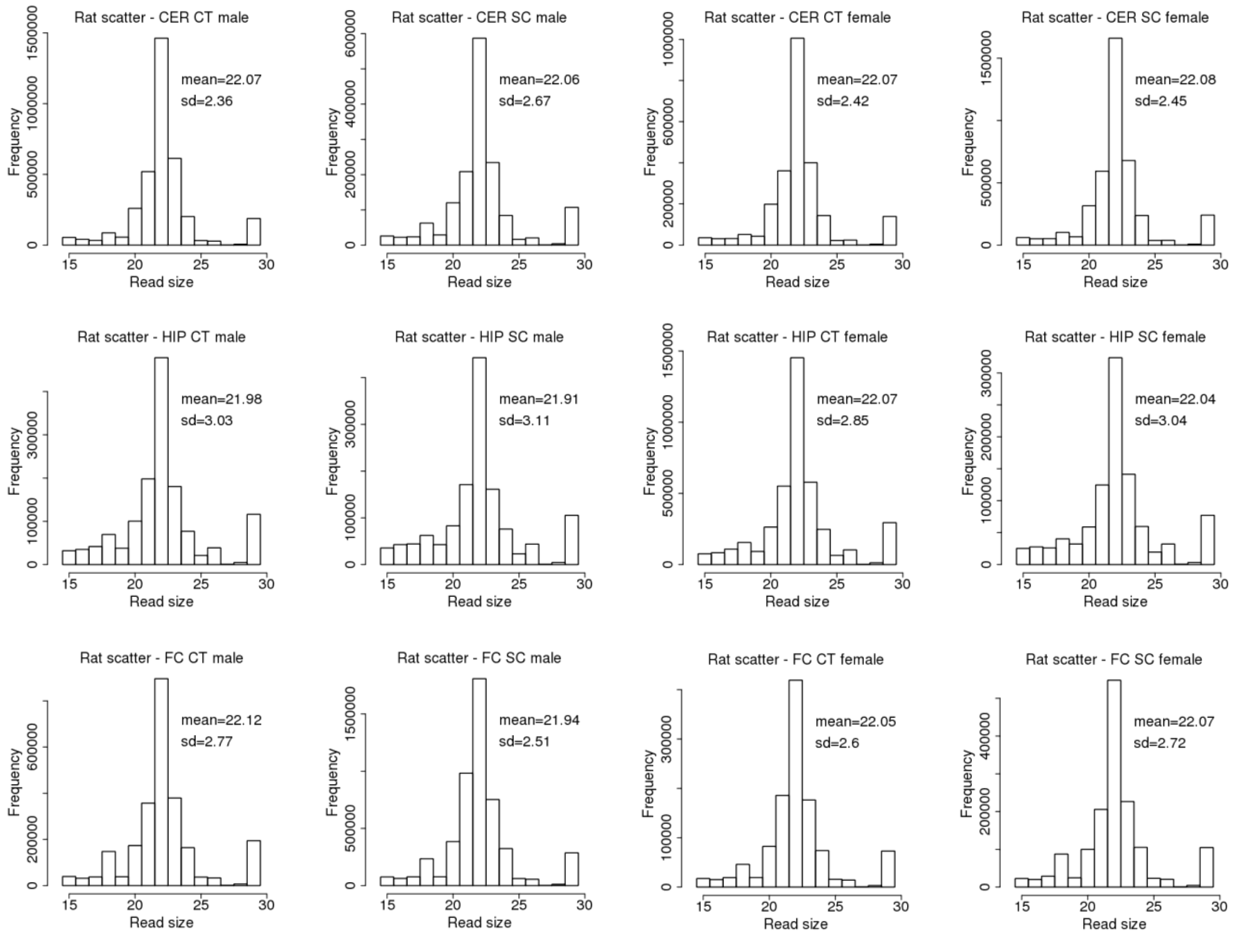

Figure S1. ncRNA read size distribution. Y axis shows the number of the reads, while X-axis shows the size of the ncRNA reads in various regions of the brain in male and female rats.

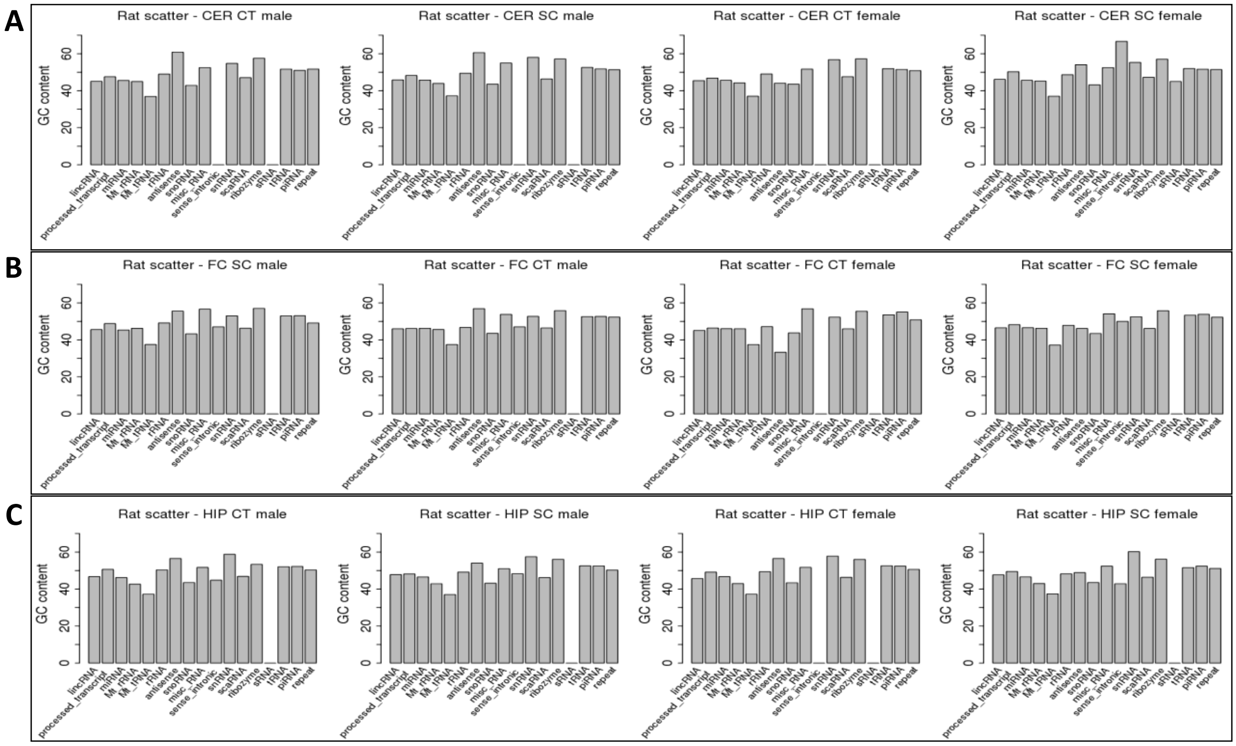

Figure S2. GC content in reads mapping to different ncRNAs in CER (A), FC (B) and HIP (C) of control and scatter-irradiated male and female rats. The Y-axis shows the percentage of GC nucleotides, while X axis shows mapping to specific ncRNAs.

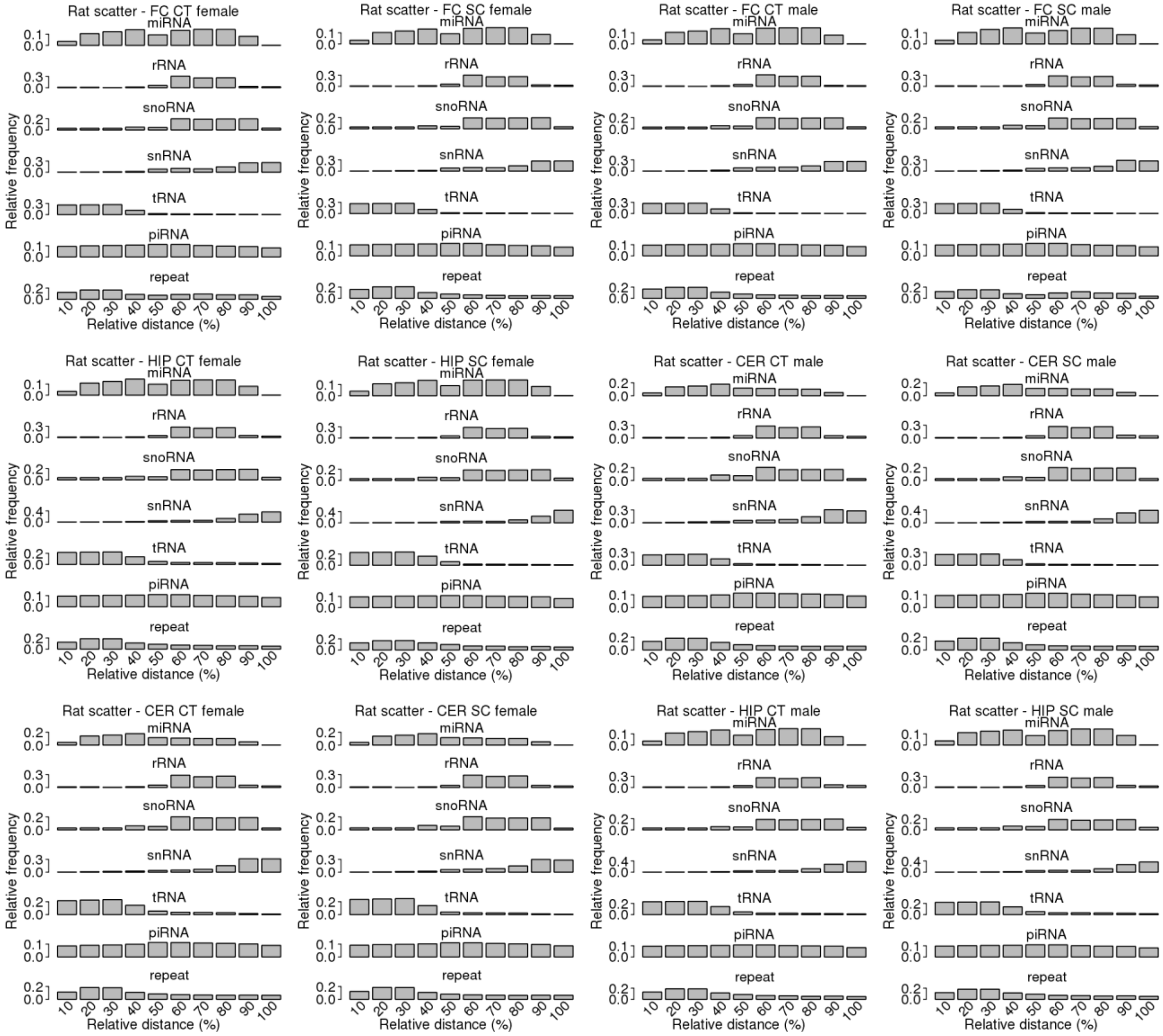

Figure S3. Analysis of reads distribution across the whole length of ncRNAs. The entire length of each ncRNA was taken as 100% and then divided into bins of 10% each, with 0 to 10% representing the first 10% of the ncRNA's length starting from the 5′ end. The data are shown as the relative frequency of reads (calculated from three biological replicates) in a specific bin calculated by dividing the frequency of reads in a specific bin by all reads mapping to the entire length of ncRNA.

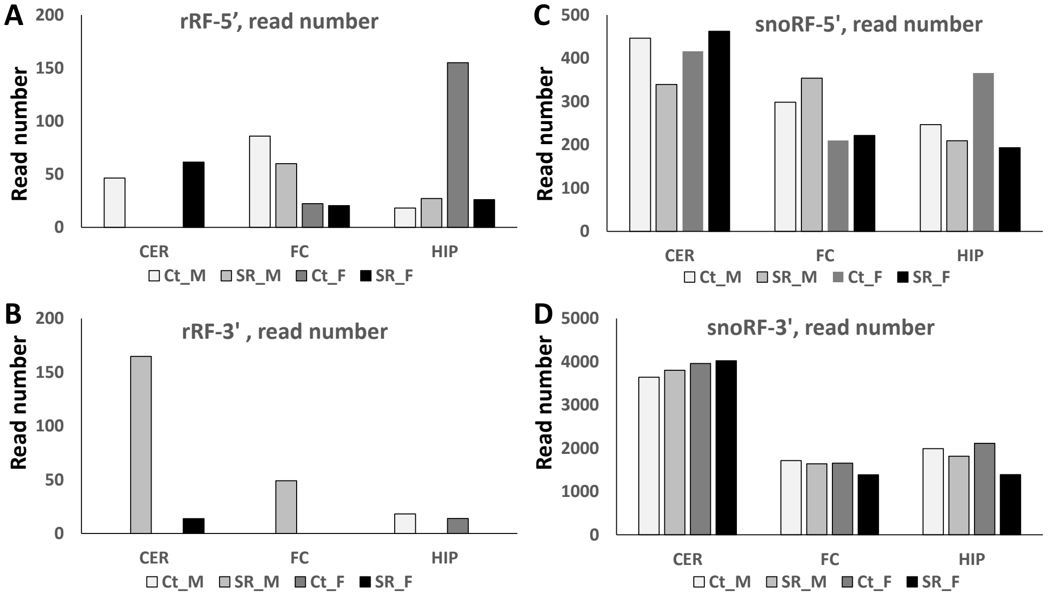

Figure S4. Number of rRF and snoRF reads mapping to the 5′ or 3′ ends of rRNA or snoRNAs. A. Number of rRF-5′ reads in various brain regions of male and female rats. B. Number of rRF-3′ reads in various brain regions of male and female rats. C. Number of snoRF-5′ reads in various brain regions of male and female rats. D. Number of snoRF-3′ reads in various brain regions of male and female rats.

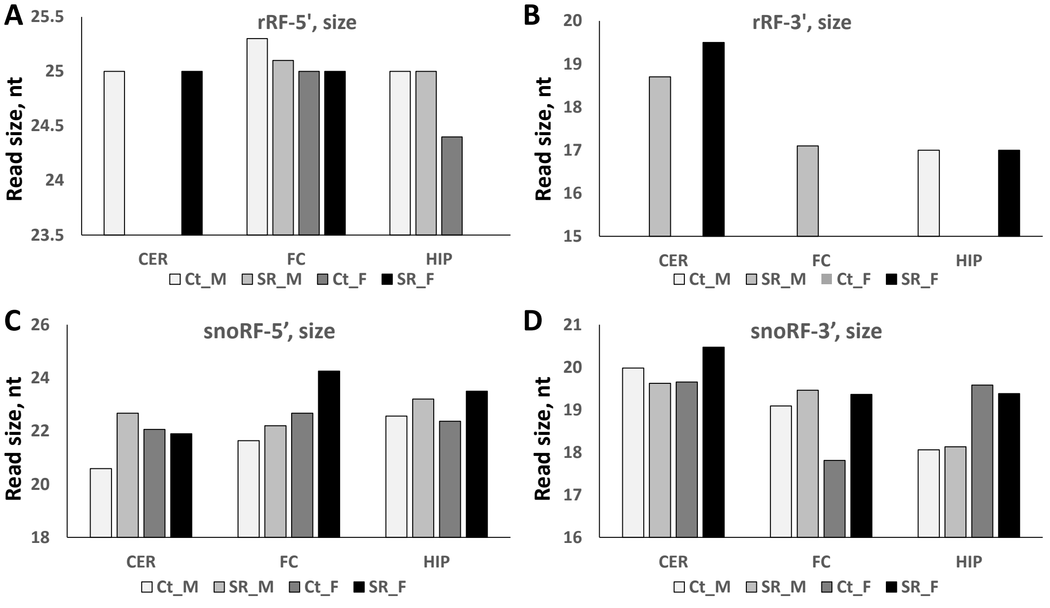

Figure S5. Size of rRF and snoRF reads mapping to the 5′ or 3′ ends of rRNA or snoRNAs. A. Size of rRF-5′ reads in various brain regions of male and female rats. B. Size of rRF-3′ reads in various brain regions of male and female rats. C. Size of snoRF-5′ reads in various brain regions of male and female rats. D. Size of snoRF-3′ reads in various brain regions of male and female rats.

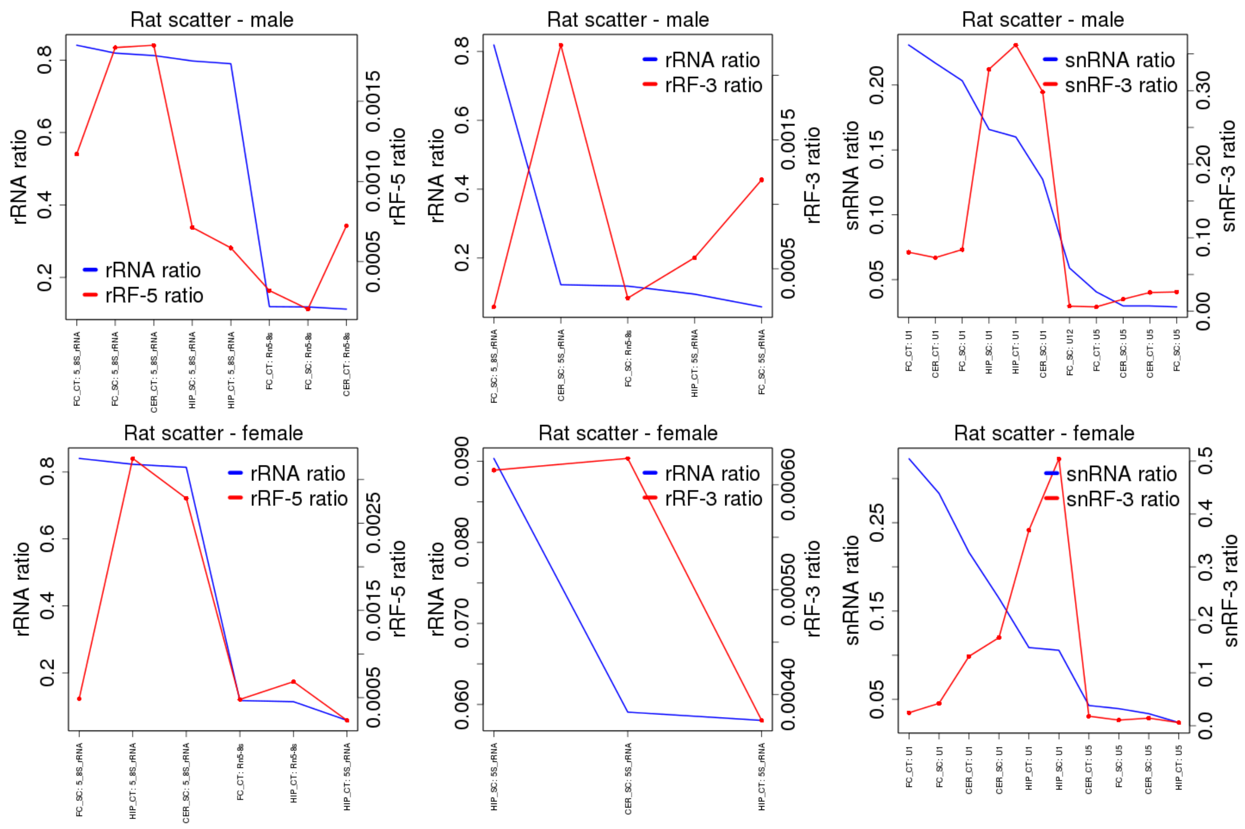

Figure S6. rRF and snRF enrichment from rRNA and snRNA in various brain regions. Upper panel – changes in male rats, lower panel – changes in female rats. The Y-axis shows in blue rRNA or snRNA ratios, and in red, rRF and snRF ratio for 5′ and 3′ – end mapping molecules. When the rRFs and snRFs peaks are larger than rRNA and snRNA peaks, there is an enrichment, while when it is lower, there is underrepresentation.
